# Identification and validation of neuroinflammation related lncRNA PVT1 with transcriptome-wide analysis in cerebral ischemia-reperfusion injury

**DOI:** 10.3389/fneur.2026.1796559

**Published:** 2026-04-29

**Authors:** Xiaochan Xiao, Huodan Yu, Haichuan Zhou, Wenjian Lu, Yanping Zeng, Qianxue Chen

**Affiliations:** 1 Department of Critical Care Medicine, Renmin Hospital of Wuhan University, Wuhan, China; 2 Department of Neurosurgery, Renmin Hospital of Wuhan University, Wuhan, China; 3 Renmin Hospital of Wuhan University, Wuhan, China; 4 The First Clinical College, Hubei University of Medicine, Shiyan, China; 5 Department of Neurology, Renmin Hospital of Wuhan University, Wuhan, China

**Keywords:** cerebral ischemia/reperfusion injury, ischemic stroke, lncRNA PVT1, neuroinflammation, T cells

## Abstract

**Background:**

The cerebral ischemia/reperfusion injury (CIRI) is an essential pathological process of ischemic stroke (IS). Secondary neuroinflammation exacerbate neuronal damage following CIRI. To identify long non-coding RNAs (lncRNAs) implicated in neuroinflammation subsequent to CIRI would significantly advance the development of potential therapeutic interventions.

**Methods:**

Through comprehensive analysis of whole-genome RNA-seq profiles in focal ischemic mice models, we identified differentially expressed genes utilizing Gene Ontology term enrichment, Kyoto Encyclopedia of Genes and Genomes pathway analysis, and gene set enrichment analysis. We further implemented immune cell infiltration deconvolution, constructed protein–protein interaction networks, and performed co-expression network analysis for lncRNA screening. Subsequently, we established the mice model with lncRNA PVT1 knockdown prior to CIRI induction. Quantitative assessment of cytokine levels was conducted using enzyme-linked immunosorbent assay, while morphological alterations were evaluated through hematoxylin–eosin staining. And T cell infiltration in cerebral tissues was detected with immunofluorescence analysis.

**Results:**

Enrichment analysis demonstrated that differentially expressed mRNAs were implicated in neuroinflammation following cerebral ischemic. Through immune deconvolution analysis, we observed a increased levels in the CD4 + and CD8 + T cells proportion of cerebral ischemic groups compared with control groups. It identified five hub lncRNAs (AI662270, AU020206, Gm20667, PVT1 and Mir142hg) exhibiting significant correlations with the expression of proinflammatory factors. Notably, PVT1 demonstrated the strongest correlation coefficient with pro-inflammatory factor mRNA expression levels. The vivo experimental validation revealed aberrantly elevated PVT1 expression following CIRI. Importantly, PVT1 knockdown substantially ameliorated CIRI through the reduction of activated T cell infiltration and pro-inflammatory cytokine secretion.

**Conclusion:**

The identified lncRNA PVT1 correlated with the activated T cell infiltration and pro-inflammatory cytokine secretion, which could be treatment target for neuroinflammation in CIRI.

## 1 Introduction

Ischemic stroke (IS), a prevalent acute cerebrovascular disorder, represents one of the leading global causes of mortality and long-term disability, which occurs when major cerebral arteries undergo thromboembolism or occlusion (1). Thrombolytic therapy utilizing tissue plasminogen activator represents the effective treatment modality presently, which is constrained by a narrow therapeutic time window (2). Following the restoration of cerebral blood supply through thrombolytic intervention, cerebral tissue is subjected to severe cerebral ischemia-reperfusion injury (CIRI) (3). CIRI represents a pathological process that exacerbates severe inflammatory responses and neural damage following the restoration of blood perfusion (4). These pathophysiological alterations ultimately culminate in acute apoptosis and necrosis of ischemic cerebral tissue, thereby including secondary brain injury (5). The suppression of inflammatory mediator has been shown to mitigate secondary CIRI (6). Consequently, the development of strategies to alleviate CIRI remains a clinical significance.

Long non-coding RNAs (lncRNAs) are characterized as transcripts exceeding 200 nucleotides in length that lack protein-coding functionality (7). LncRNA plays a crucial regulatory roles in gene transcriptional processes, particularly in mediating cardio-cerebrovascular protective mechanisms and orchestrating immune cell activation pathways (8). It has demonstrated that lncRNAs influence CIRI and modulate gene expression through the regulation of neuroinflammation and microglial apoptosis (9). For instance, small nucleolar RNA host gene 14 and taurine-upregulated gene 1 have been demonstrated to significantly influence cellular apoptosis and inflammatory responses in CIRI (10). A comprehensive elucidation of the molecular mechanisms underlying CIRI would facilitate the development of innovative strategies for the diagnosis and therapeutic intervention. Nevertheless, the research on lncRNA in modulating inflammatory responses during CIRI remains limited.

For our study, it identified the lncRNA plasmacytoma variant translocation 1 (PVT1) as being associated with neuroinflammation following CIRI. PVT1, located within the telomeric region of chromosome 8 adjacent to the c-Myc gene, was initially characterized by Guan et al. (11). It has indicated that down-regulated PVT1 may enhance spatial cognitive function and memory retention, while concurrently attenuate neuronal degeneration and apoptotic processes in epileptic conditions (12). However, the specific role of PVT1 in CIRI remained to be elucidated. We employed comprehensive bioinformatic analysis and functional validation to systematically screen lncRNA candidates implicated in neuroinflammation. Our study initially validated the role of lncRNA PVT1 in neuroinflammation following CIRI, revealing its potential as a therapeutic target for modulating post-ischemic inflammatory cascades.

## 2 Materials and methods

### 2.1 Whole-genome transcriptomic data processing

The raw sequencing data (GSE137482) in SRA format were downloaded from the NCBI Gene Expression Omnibus (GEO, https://www.ncbi.nlm.nih.gov/gds/). And in GSE137482, there contains 24 samples, which had 12 samples of the 3 and 18 month-old C57BL/6 mice, respectively. Subsequently, we downloaded the “gencode.vM31.annotation.gtf” file from the GENCODE website^1^ to annotated gene symbols by “tidyverse” R package in Rstudio. And the “rlogTransformation” function of “DESeq2” R package was employed to remove the batch effect and make the expression data normalized.

### 2.2 Identification of differentially expressed genes

The Deseq2 R package was employed for normalization and DEGs analyses (13). Redundant gene entries were eliminated, with mean expression values retained. Statistical significance was determined using the following criteria: *p* < 0.05, FDR-adjusted q-value <0.05, with minimum expression changes of absolute log2 fold change ≥1 for mRNAs or ≥0.585 for lncRNAs.

### 2.3 Functional and pathway enrichment analyses

Functional annotation analysis was performed using clusterProfiler (Bioconductor R package) to conduct Gene Ontology (GO), Kyoto Encyclopedia of Genes and Genomes (KEGG)^2^ and Gene Set Enrichment Analysis (GSEA) (14). The GO framework was employed to classify gene functions into three ontologies: biological process (BP), cellular component (CC), and molecular function (MF). Pathway analysis was performed using KEGG and GSEA databases with a significance threshold of *p* < 0.05. BP term interaction networks were visualized using Cytoscape (15) with the ClueGO plugin (16). The STRING database^3^ was employed to investigate protein–protein interaction networks.

### 2.4 Co-expression network of lncRNA-mRNAs

The mRNA gene symbols associated with T cell activation regulation were retrieved from the MSigDB database.^4^ The MCODE tool in Cytoscape was applied to identify central hub genes among the T cell activation regulatory mRNAs, and these key genes were incorporated into the lncRNA-mRNA co-expression network construction. The relative abundance of various immune cell populations in the samples was assessed with seq-immuCC (17). The Pearson correlation coefficient was computed between each differentially expressed lncRNA and these mRNAs based on their expression values. Strong correlations were defined as those with the absolute value of a correlation ≥0.95 and *p* < 0.05 for all analyzed lncRNA-mRNA pairs (either positive or negative).

### 2.5 Animal

The male C57BL/6 J mice were provided by Shulai Bao Biotechnology Co. Ltd. and subsequently housed in specific pathogen-free facility at the Experimental Animal Center of Renmin Hospital of Wuhan University. The animals were maintained under a 12-h light–dark cycle, temperature conditions of 23 ± 2 °C, and relative humidity of 60%, with food and water available at all times. Ethical clearance for this research was obtained from the Ethics Committee at Renmin Hospital of Wuhan University (Reference No. WDRM-2025-K073), with strict adherence to AAALAC policies and ARRIVE reporting standards. Mice were randomly allocated to experimental groups with matched body weights and maintained under isoflurane anesthesia (4% induction, 2% for maintenance) during surgery.

### 2.6 Silencing of the lncRNA PVT1

Knockdown of PVT1 was achieved using 2′-OMe antisense oligonucleotides (ASOs) synthesized by RiboBio (RiboBio Co., Ltd., Guangzhou, China). The ASOs targeting PVT1 (sequence: 5′-CTTTTAGTATCCTGAAATGTG-3′) was administered via intracerebroventricular injection. Mice were anesthetized with 1% pentobarbital sodium (60 mg/kg, intraperitoneal) and then placed in a stereotactic apparatus. A cranial puncture was performed at the following coordinates: x = 1.5 mm lateral to the sagittal suture, y = −2.0 mm posterior to bregma, and z = 2.5 mm in depth. Either PVT1 ASO (2 nmol, 2 μL) or scrambled ASO-NC (negative control) was injected intracerebroventricularly once weekly for two consecutive weeks prior to CIRI induction.

### 2.7 Middle cerebral artery occlusion model

The MCAO model was employed to simulate CIRI. Following anesthesia induction, a midline cervical incision was performed to expose the common carotid artery, the external carotid artery was ligated, and the internal carotid artery was subsequently isolated. A nylon monofilament with a silicone-coated tip was advanced into the internal carotid artery via the external carotid artery until mild resistance was encountered. Cerebral blood flow was occluded for 60 min, after which the monofilament was withdrawn to facilitate cerebral reperfusion. The ischemic penumbra tissue samples were harvested for further experimental analyses after the reperfusion period.

### 2.8 Experiment 1

Expression levels of 5 lncRNAs were quantitatively analyzed at 24 h post-CIRI using quantitative polymerase chain reaction (qPCR). Mice were randomly assigned to receive either sham operation or CIRI groups.

### 2.9 Experiment 2

Mice were randomly assigned to the sham-operated group, CIRI group, CIRI+ASO-NC group and CIRI+ASO-PVT1 group. Subsequent analyses included qPCR, enzyme-linked immunosorbent assay (ELISA), 2,3,5-triphenyl tetrazolium chloride (TTC) staining, brain water content (BWC), hematoxylin–eosin (HE) staining, and immunofluorescence (IF) staining.

### 2.10 Experiment 3

Three experimental groups were established through random mice allocation: CIRI only, CIRI + negative control ASO group, and CIRI + PVT1 ASO group, to systematically assess the effect of PVT1 knockdown on neurological functional evaluation following CIRI induction.

### 2.11 Neurological function evaluation

Neurological function was evaluated using the Modified Neurological Severity Score (mNSS) (18) at 24, 48, and 72 h post-CIRI. The scoring system ranged from 0 (normal) to 14 (maximal deficit), with elevated scores corresponding to greater neurological dysfunction. Two blinded evaluators independently performed all assessments.

### 2.12 TTC staining

After CIRI, following deep anesthesia, mice underwent transcardial perfusion with PBS. The cerebral tissue was promptly excised, flash-frozen at −20 °C for 20 min, and sectioned into 2 mm thick coronal slices. The tissue specimens were then homogenized with 2% TTC (Jiancheng Biotech) solution and incubated under dark conditions at 37 °C for 30 min. To minimize the confounding effect of brain edema, Infarct volume was calculated through the following formula: infarct volume = [(volume of contralateral hemisphere – volume of non-ischemic ipsilateral hemisphere)/volume of contralateral hemisphere] × 100%.

### 2.13 BWC analysis

The cerebral tissue was obtained post-CIRI. Both ipsilateral and contralateral hemispheres were immediately weighed for wet weight measurement, then dried at 75 °C for 48 h to obtain dry weight values. The cerebral edema index was determined by the formula: [(wet weight − dry weight)/wet weight] × 100%.

### 2.14 qPCR

Using TRIzol reagent (Invitrogen™ TRIzol™, Thermo Scientific, United States), total RNA was purified, and 1 mg of aliquots were subjected to cDNA synthesis with RT Master Mix (Takata Bio, Dalian, China). qPCR analysis was conducted using SYBR Green chemistry with gene-specific primers (Supplementary Table S1). The thermal cycling program was set as: 95 °C for 5 min; 40 × (95 °C for 10 s, 60 °C for 60 s). The 2^−ΔΔC^ method was employed for relative quantification of target lncRNAs, normalized to Gapdh expression.

### 2.15 ELISA

After CIRI, the experimental mice were subjected to anesthesia followed by transcardial perfusion with phosphate-buffered saline (PBS, pH 7.4, 4 °C). The infarcted cerebral hemisphere was promptly excised, homogenized in 200 mg/mL physiological saline (0.9%), and centrifuged at 12,000 rpm for 20 min at 4 °C. The resulting supernatant was aliquoted and cryopreserved at −80 °C for subsequent analyses. Quantitative determination of inflammatory cytokines, including interleukin 6 (IL-6), interleukin 1 beta (IL-1β), interleukin 4 (IL-4), and interleukin10 (IL-10) in the brain tissue lysates was performed using commercially ELISA kits (#88-7064-22, #88-7013-22, #88-7044-22, and #88-7105-22, Invitrogen, Thermo Scientific, United States), we adhered to the manufacturer’s protocol. The cytokine concentrations were quantified through standard curve analysis based on absorbance measurements.

### 2.16 H&E staining

Following anesthesia, transcardial perfusion (4 °C PBS, pH 7.4) and paraformaldehyde fixation (4%) were performed on all experimental mice. Brains were excised and fixed in 4% formaldehyde (24 h, 4 °C). The samples were dehydrated through an ethanol gradient and paraffin-embedded. Embedded specimens were sectioned at a thickness of 4 μm using a microtome. The obtained sections were then deparaffinized and rehydrated through a descending ethanol series, followed by HE staining (Cat. #G1005, Servicebio, Wuhan, China) in accordance with the manufacturer’s protocol.

### 2.17 IF staining

Following perfusion, the mice were euthanized, and the brain tissues were processed by 4 °C overnight fixation in 4% paraformaldehyde. Subsequently, the tissues underwent stepwise dehydration in 10, 20, and 30% sucrose solutions at 4 °C. Brains were embedded in optimal cutting temperature, cryosectioned at 10 μm, and used for IF analysis.

Brain tissue sections and cell slides were initially rinsed with PBS and subsequently fixed with PFA at ambient temperature for 10 min. The samples were blocked for 1 h at room temperature with 1% bovine serum albumin (BSA; AMRESCO, United States), 0.1% Triton-100 (Sigma) and 10% goat serum (Beyotime, China). The samples were probed overnight at 4 °C with anti-CD4 (1:1000, Abcam) and anti-CD8 (1,500, Abcam) primary antibodies. Secondary antibodies (Alexa Fluor-488/594/647; Jackson ImmunoResearch, United States) were applied to samples for 1-h dark incubation. After thorough PBS washing, the tissue sections were mounted using CC/MOUNT mounting medium (Sigma). Fluorescence Imaging was performed using either an Olympus MX51 fluorescence microscope (Olympus MX51, Japan) or a FluoView FV3000 confocal laser scanning microscope (Olympus Corporation).

### 2.18 Statistical analysis

Data were statistically evaluated through R software (version 4.0.2). Visualization of differential expression patterns was achieved through the “EnhancedVolcano” package. Hierarchical clustering heatmaps were generated with the “pheatmap” package. GO analysis and chord diagrams were implemented via the “Goplot” package. Survival analysis was performed with the “survminer” package. Correlation matrices were computed and visualized using the “corrplot” package. Shapiro–Wilk normality test was applied to continuous data. Parametric and non-parametric comparisons utilized Student’s *t*-test and Mann–Whitney U test, respectively. Continuous variables are expressed as mean ± standard deviation (SD) or standard error of the mean (SEM). A significance threshold of *p* < 0.05 was applied for all statistical comparisons.

## 3 Results

### 3.1 Sequence data summary and DEG analysis

**Figure 1:**
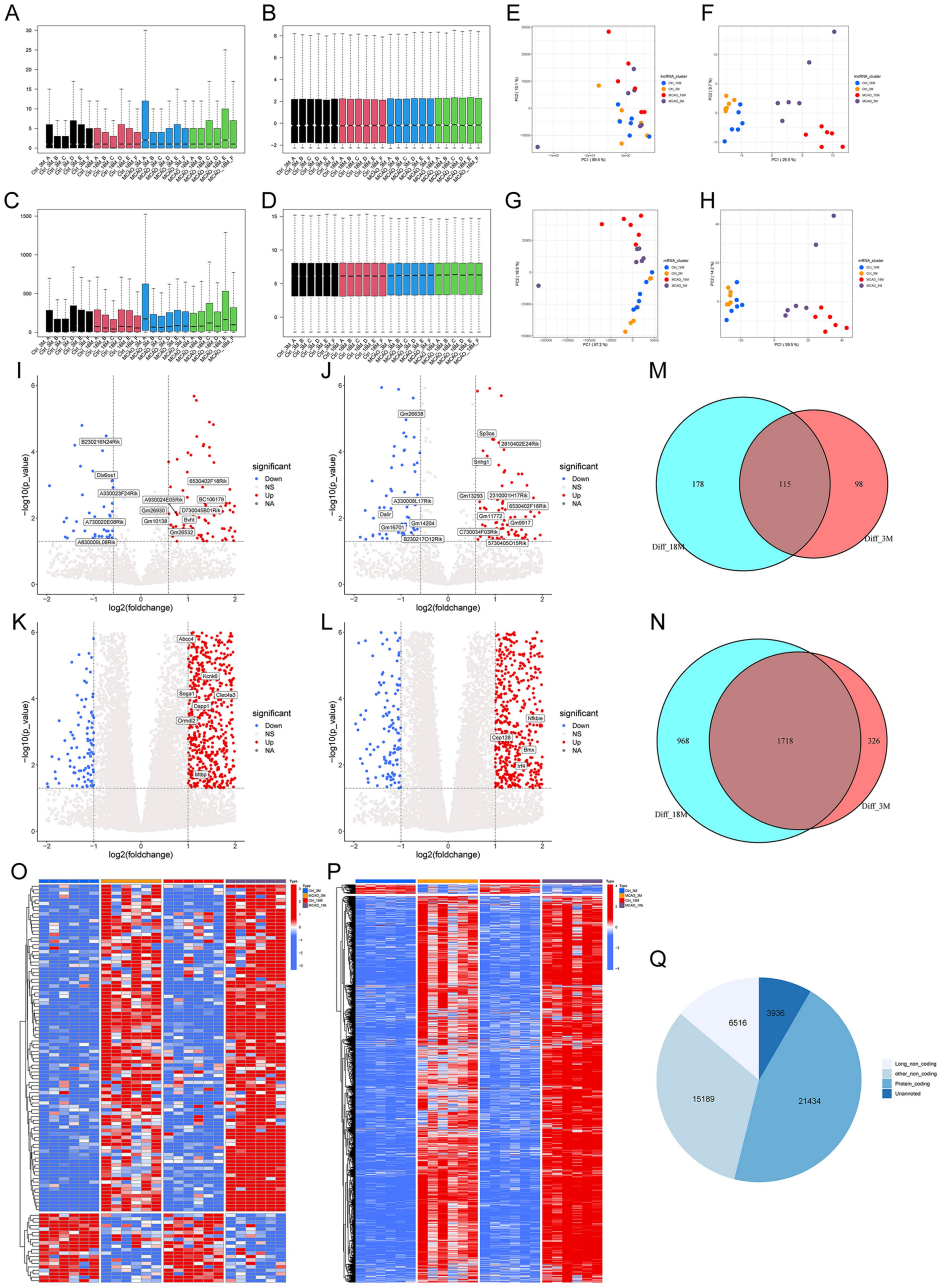
Sequence data summary and DEG analysis. **(A,C)** Assessment of batch effects on lncRNA and mRNA expression profiles across the four groups. **(B,D)** Elimination of batch effects following correction of the tissue samples. **(E–H)** Principal component analysis of lncRNA and mRNA datasets. **(I–L)** Volcano plots displaying genome-wide differential expression. **(M,N)** Venn diagrams showing overlaps of differentially expressed mRNAs and lncRNAs at different time points. **(O,P)** Heatmaps depicting the expression patterns of selected differentially expressed genes over time. **(Q)** Pie chart categorizing all annotated gene types.

The boxplot showed the batch effects between 4 groups in the expression levels of lncRNA and mRNA, respectively (Figures 1A,C). Subsequently, Figures 1B,D respectively exhibited the normalized data of lncRNA and mRNA, which indicated that the batch effects between these 4 groups had been removed. Using the mouse genome (mm10) whole-genome alignment, principal component analysis (PCA) indicated that these 24 samples could not be distinguished into 4 distinct groups before normalization (Figures 1E,G), while after standardization, the samples could be significantly distinguished (Figures 1F,H). Comparative analysis of transcriptome profiles across age-matched control and MCAO cohorts, the volcano maps showed the differently expressed lncRNAs (Figures 1I,J) and mRNAs (Figures 1K,L), respectively. Then, we take intersections between 3 and 18 month-old (3 M and 18 M) mice groups of lncRNA and mRNA, respectively. And in Figure 1M, the Venn diagram exhibited there were 115 intersected differently expressed lncRNAs between 3 M and 18 M groups, which was also visualized by heatmap (Figure 1O), while 213 differently expressed lncRNAs in 3 M groups and 293 differently expressed lncRNAs in 18 M groups. Figure 1N showed that 1718 intersected DEGs between 3 M and 18 M groups, which was also visualized by heatmap (Figure 1P), while 2044 DEGs in 3 M groups and 2,686 DEGs in 18 M groups. Of note, there were significantly more up-regulated genes than that of down-regulated genes after MCAO. Furthermore, the pie chart exhibited the types of all annotated gene symbols, indicated a total of 43,139 genes were mapped and 21,434 mRNA genes and 6,516 lncRNA genes (Figure 1Q).

### 3.2 GO term enrichment and KEGG pathway analysis

**Figure 2:**
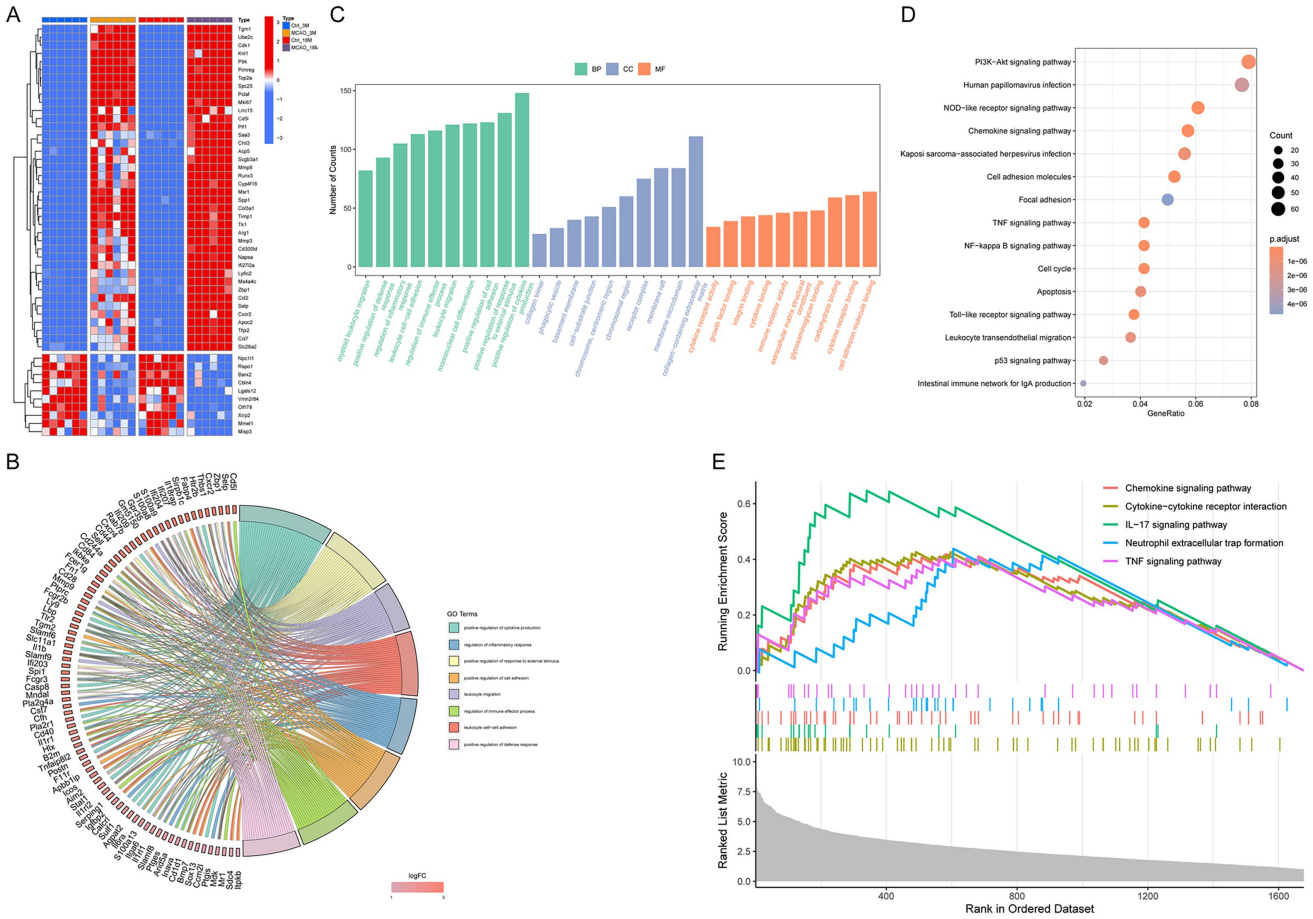
GO term enrichment and KEGG pathway analysis. **(A)** Expression patterns of the top 40 upregulated and top 10 downregulated DEGs are shown in a heatmap. **(B)** GO-Chord plot visualizing the relationship between mRNAs and their enriched biological process terms. **(C)** Significantly enriched terms in biological process, cellular component, and molecular function categories. **(D)** KEGG pathway enrichment analysis conducted with the DAVID tool. **(E)** GSEA plot displaying enriched pathways.

Heatmap visualization of the top 40 upregulated and top 10 downregulated protein-coding DEGs (Figure 2A) was followed by systematic functional exploration using GO and KEGG pathway enrichment frameworks. Upregulated mRNAs demonstrated significant enrichment in immune-related biological processes, including cytokine production modulation, inflammatory response regulation, immune effector activation, and defense response potentiation, along with leukocyte/monocyte migration pathways. Concurrently, GO cellular component and molecular function analyses further corroborated their association with inflammatory mechanisms (Figures 2B,C). Integrated KEGG-GSEA profiling identified significant in TNF-mediated signaling and chemokine pathway activation, as illustrated in Figures 2D,E. Functional enrichment analyses revealed significant association between DEGs and post-MCAO inflammatory mechanisms.

### 3.3 Co-expression analysis of lncRNA correlated with inflammation

**Figure 3:**
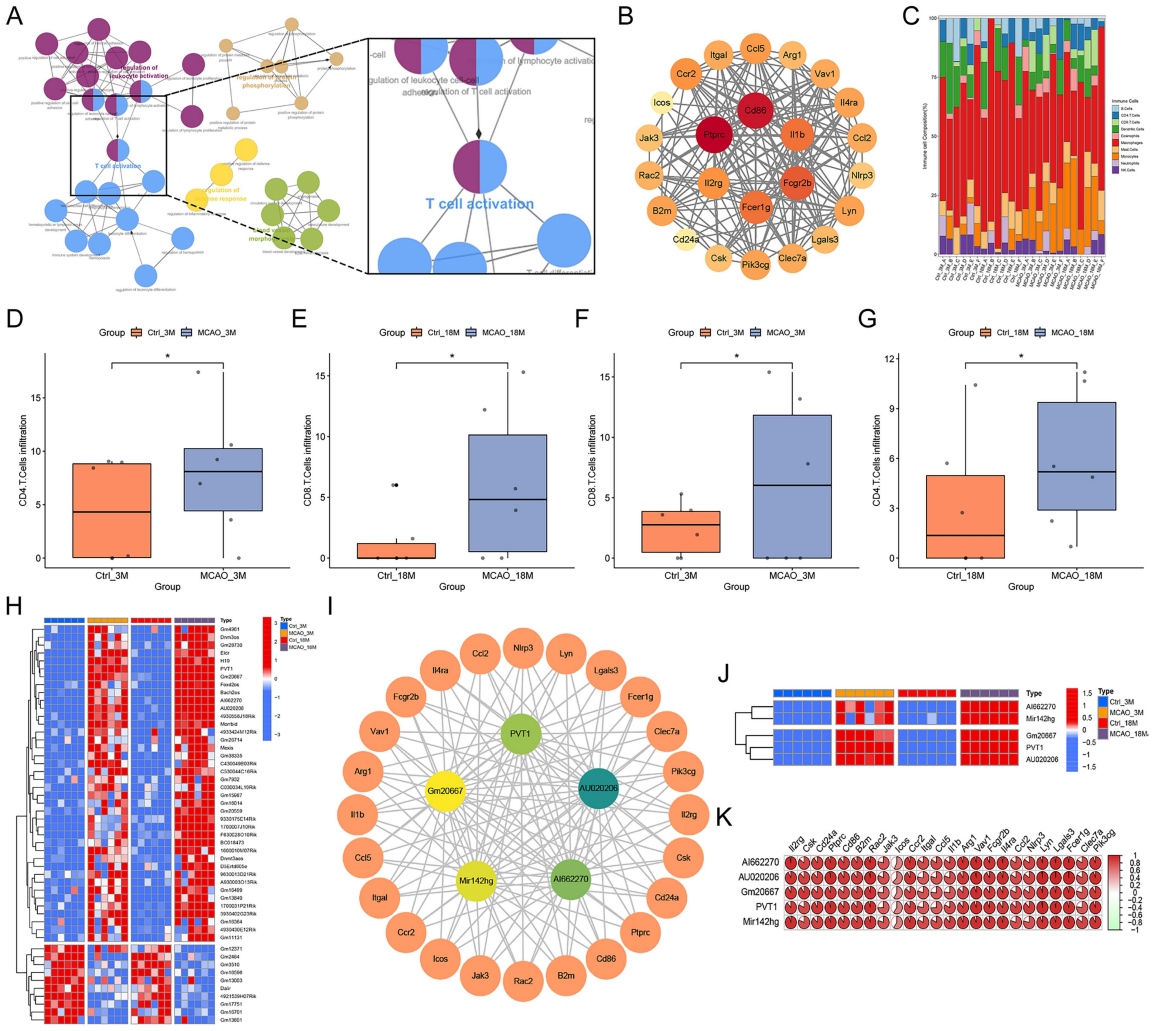
Co-expression analysis of lncRNA correlated with inflammation. **(A)** GO term network with a focus on T cell activation pathways (ClueGO). **(B)** PPI network of DEG-encoded hub proteins for T cell activation. **(C)** Relative abundance of immune cell types in each sample, as estimated by seq-ImmuCC. **(D–G)** Elevated proportions of CD4^+^ and CD8^+^ T cells in the MCAO group. **(H)** Heatmap of the top 40 up- and 10 down-regulated lncRNAs. **(I,J)** Top five lncRNAs associated with T cell activation. **(K)** Quantitative depiction of the co-regulatory network between five lncRNAs and 24 mRNAs using a pie chart matrix.

To identify the lncRNA that regulated inflammation, we first used clueGO to perform network analysis on the enriched GO terms. The results showed that inflammation was closely related to the activation of T cells (Figure 3A). Then we uploaded those genes related to T cell activation in Cytoscape software, screened out the hub genes by MCODE function and the PPI network was established, encompassing pivotal mRNAs associated with T cells activation (Figure 3B). Immune cell proportion analysis using seq-immuCC revealed T cell subset variations in post-MCAO mice brains through computational deconvolution. MCAO groups displayed higher levels in the CD4^+^ and CD8^+^ T cells proportion compared with control groups both in 3 M and 18 M groups (Figures 3C–G). Hierarchical clustering in Figure 3H delineated the expression patterns of top-ranking lncRNAs, with 40 exhibiting marked upregulation and 10 showing pronounced downregulation across experimental cohorts. Ultimately, we conducted a co-expression analysis on the transcript expression values of these differential key mRNAs, related to T cell activation, and differential lncRNAs, and we identified the 5 lncRNAs with the strongest correlation coefficients (|cor| > 0.95 and *p* < 0.05) from the matrix of long non-coding DEGs (Figures 3I,J). Figure 3K quantitatively illustrates 5 lncRNAs and 24 mRNAs co-regulatory relationships through the pie chart matrix.

### 3.4 Verification of lncRNAs related to T cells activation

**Figure 4:**
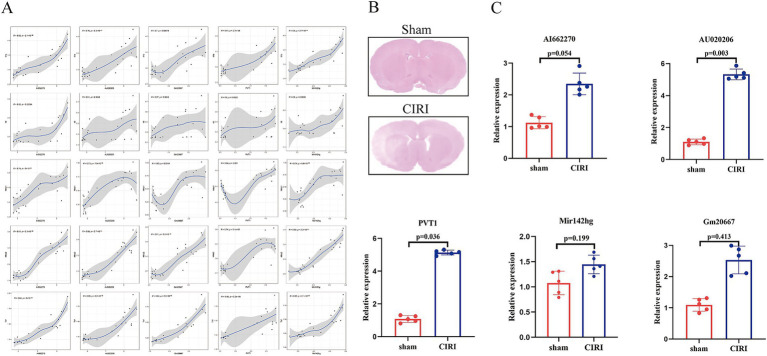
Verification of lncRNAs related to T cells activation. **(A)** Correlation between five lncRNAs (AI662270, AU020206, Gm20667, PVT1, Mir142hg) and pro-inflammatory factors. Axes represent normalized expression levels; correlation coefficients and *p*-values are shown. **(B)** Representative HE-stained brain tissue sections. **(C)** qPCR validation of lncRNA expression. AU020206 and PVT1 were significantly upregulated in the CIRI group. Data are presented as mean ± SEM; * *p* < 0.05 versus sham group (*t*-test), (*n* = 5).

During the initial phase of CIRI, activated T cells can secrete an array of pro-inflammatory factors, promote neuroinflammation and aggravate brain damage (19). We analyzed the correlation between the expression levels of 5 candidate lncRNAs and the proinflammatory factor mRNA. The results indicated that the 5 lncRNAs, including AI662270, AU020206, Gm20667, PVT1, and Mir142hg, were positively correlated with the expression of proinflammatory cytokines (Figure 4A). To further experimentally validate related lncRNAs, we employed an established CIRI model, with subsequent transcriptional validation to identify lncRNAs at 24 h post-CIRI.

The results of the HE staining on cerebral tissues indicated that the model was successfully established (Figure 4B). Subsequently, quantification of the expression profiles showed that AI662270, Gm20667, and Mir142hg had no significant differences, while AU020206 and PVT1 were significantly increased (Figure 4C). Cross-validation of transcriptomic predictions with experimental qPCR verification revealed PVT1 as the most robustly dysregulated lncRNA, prompting its selection for further investigation.

### 3.5 PVT1 knockdown ameliorated ischemic brain insult and reduced pro-inflammatory cytokine release

**Figure 5:**
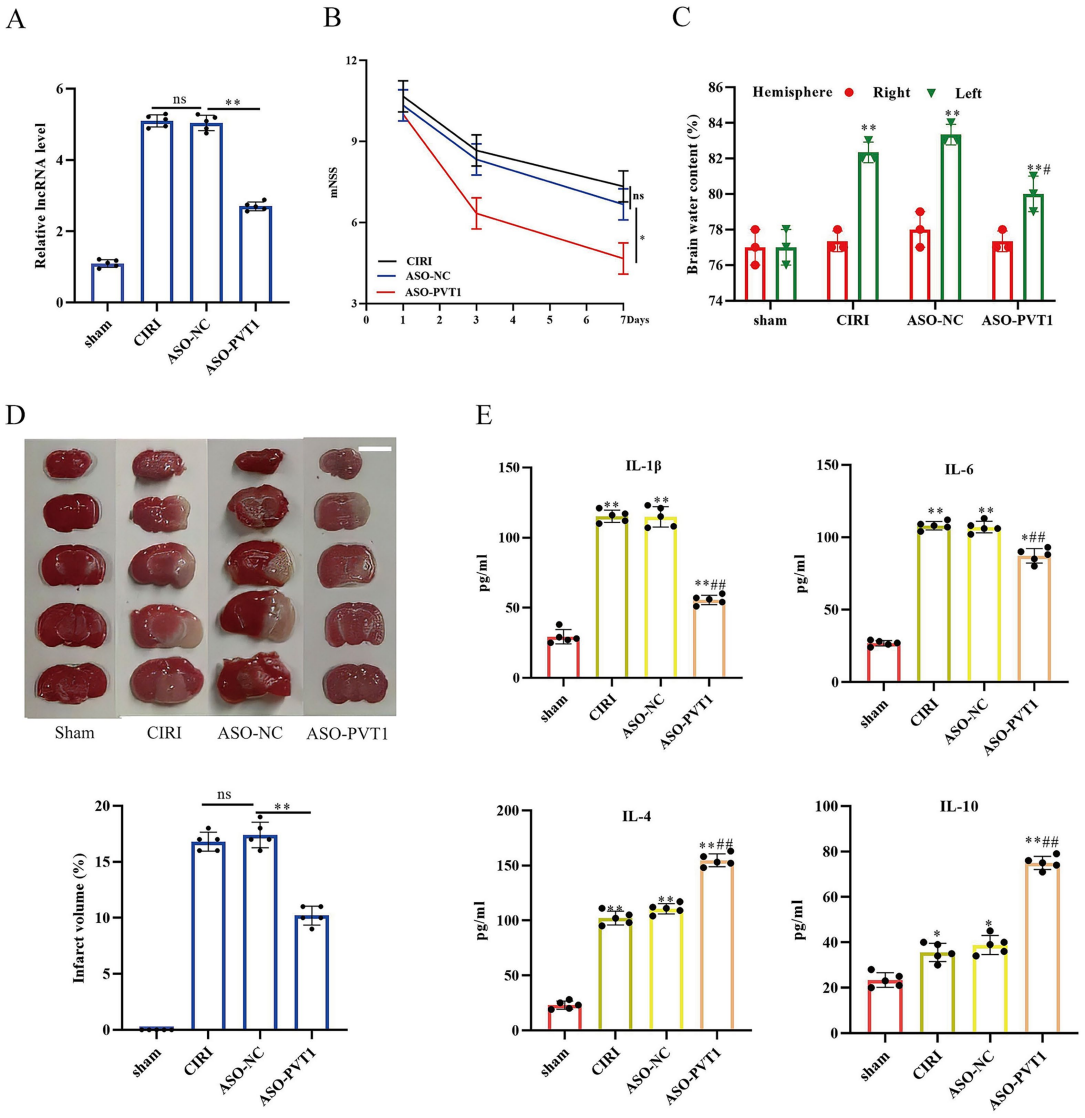
PVT1 knockdown ameliorated ischemic brain insult and reduced pro-inflammatory cytokine release. **(A)** Efficient PVT1 knockdown was verified by qPCR. Data: mean ± SEM; ** *p* < 0.01 (Mann–Whitney test). **(B)** PVT1 knockdown improved neurological function scores. **(C)** Brain water content measurement. **(D)** Representative TTC-stained brain sections from sham, CIRI, ASO-NC, and ASO-PVT1 mice at 24 h post-operation. **(E)** ELISA quantification of pro-inflammatory mediators. Data are mean ± SD; * *p* < 0.05, ** *p* < 0.01 vs. indicated groups (*t*-test) (*n* = 5).

To delineate the pathophysiological role of PVT1 at 24 h post-CIRI *in vivo*, we constructed the mice with PVT1 knockdown firstly (Figure 5A). We then rated the neurological severity scores of mice in different groups. The results showed that the neurological function of the mice was impaired significantly following CIRI, but the ASO-PVT1 group showed lower function damage compared to the CIRI and ASO-NC groups (Figure 5B). Furthermore, PVT1 knockdown mice exhibited reduced cerebral edema (Figure 5C) and diminished ischemic lesions by TTC staining (Figure 5D). We further systematically evaluated whether PVT1 knockdown regulated the levels of inflammatory factors. ELISAs determined two pro-inflammatory cytokines and two anti-inflammatory cytokines. PVT1 silencing attenuated MCAO-induced proinflammatory mediators (IL-1β; IL-6) while elevating immunoregulatory cytokines (IL-10; IL-4) (Figure 5E). These findings demonstrated that knockdown of PVT1 alleviated the inflammatory response, consequently alleviating brain injury.

### 3.6 PVT1 knockdown ameliorated CIRI and reduced the infiltration of activated T cells

**Figure 6:**
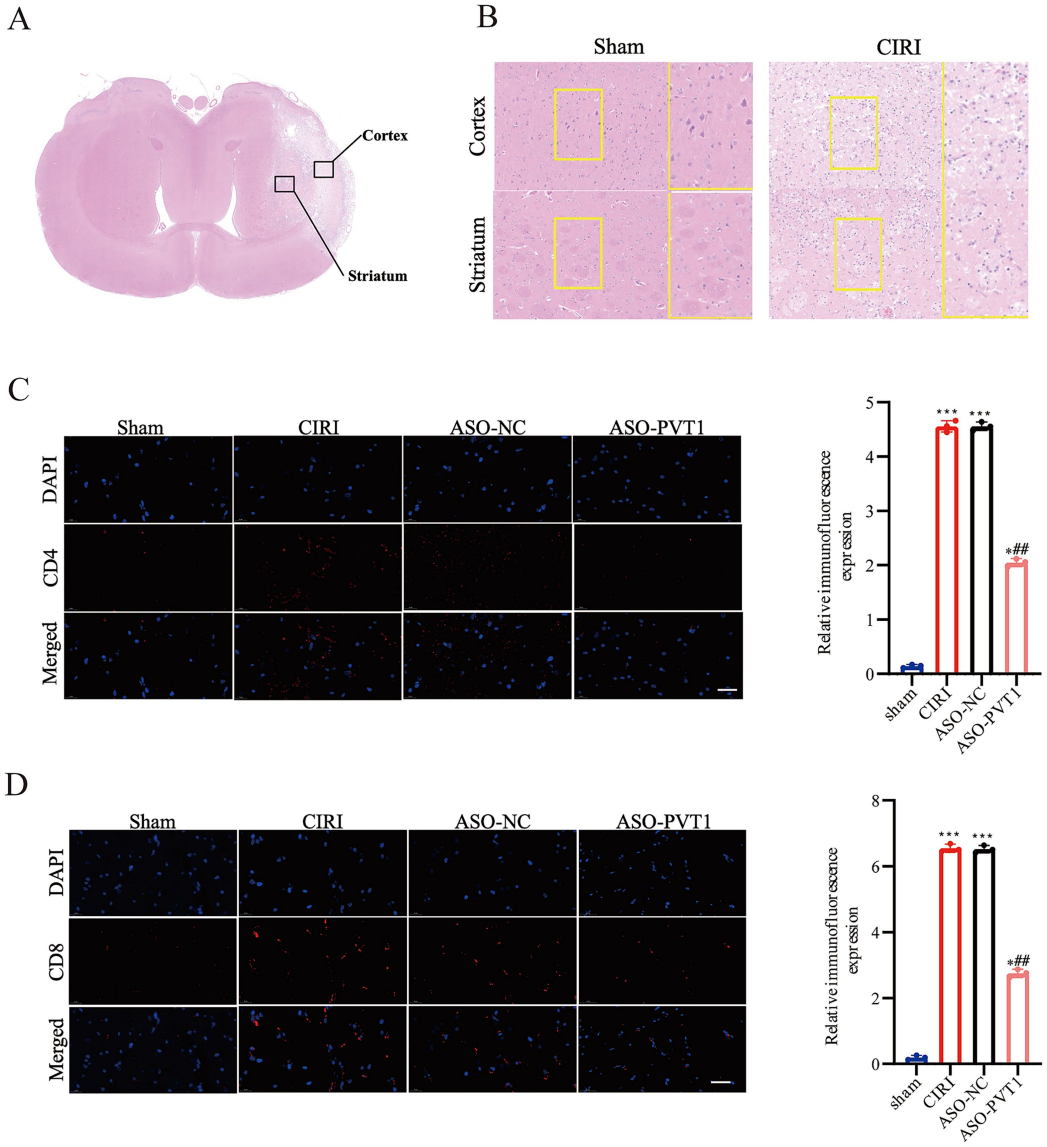
PVT1 knockdown ameliorated CIRI and reduced the infiltration of activated T cells. **(A)** HE staining of the ischemic penumbra in the cortex and striatum following CIRI. **(B)** Coronal sections were stained with H&E. **(C)** CD4 + T cell infiltration levels assessed by immunofluorescence, (*n* = 3). **(D)** CD8 + T cell infiltration levels assessed by immunofluorescence, (*n* = 3).

Subsequently, we performed microscopic examination of the pathological changes in the cerebral cortex and striatum, including the ischemic core and penumbra, at 24 h after CIRI, as shown in Figure 6A. HE staining was conducted to examine morphological alterations. Notably, comparing with CIRI and ASO-NC groups, the nuclear pyknosis and cytoplasmic vacuolation in both the cortex and striatum of mice exhibited significant reduction with PVT1 knockdown (Figure 6B). Following CIRI, CD4 + and CD8 + T cell levels were also detected across all experimental groups using IF staining. There were significantly fewer activated CD4 + T cells and CD8 + T cells in the PVT1 knockdown group comparing with the CIRI and ASO-NC groups (Figure 6C). Above results indicated that PVT1 knockdown alleviated CIRI via the reduction of activated CD4 + T cells and CD8 + T cells (Figure 6D).

## 4 Discussion

An increasing amount of evidence indicates that lncRNAs serve pivotal regulatory functions in physiological and pathological responses related to several diseases, but their roles in the pathological mechanisms and progression of CIRI remain unknown (20). In this study, multiple bioinformatics methods, including differential analysis, immune component deconvolution, GO and KEGG enrichment analysis, and lncRNA-mRNA transcriptional network modeling, were employed to identify neuroinflammation-related lncRNAs in CIRI. Ultimately, five lncRNAs (AI662270, Mir142hg, Gm20667, PVT1, and AU020206) were found to be significantly correlated with neuroinflammation in CIRI, among which PVT1 demonstrated a strong correlation with T cell-mediated neuroinflammation.

Previous studies have reported that in ischemic and hypoxic neuronal models, upregulation of PVT1 significantly reduces neuronal viability and promotes apoptosis. It exerts pro-inflammatory functions through the microRNA-24-3p/STAT3 signaling axis, thereby exacerbating pathological damage in IS (21). In the present study, we found that PVT1 plays a pro-inflammatory role during the pathogenesis of CIRI. Using a mouse model of MCAO, we demonstrated that PVT1 knockdown leads to downregulation of pro-inflammatory cytokines such as IL-1β and IL-6, as well as reduced infiltration of CD4 + and CD8 + T cells.

Although we have not directly investigated the molecular mechanisms, extensive previous findings suggest two possible mechanisms. First, PVT1 may indirectly influence T cell activation and chemotaxis by modulating the inflammatory phenotype of microglia or astrocytes. Both microglia and astrocytes participate in post-stroke immune regulation and interact with T cells to contribute to neuroinflammation (22). For instance, astrocytes can secrete IL-15, increasing the levels of CD8 + T cells and natural killer cells, thereby aggravating brain injury (23). Moreover, PVT1 has been shown to significantly enhance astrocyte activation and, through regulation of miR-186-5p and the CXCL13/CXCR5 axis, induce astrocyte activation that exacerbates neuropathic pain in rats with spinal cord injury (24). Second, PVT1 may directly regulate T cell activation and differentiation. Although we did not perform transcriptomic analysis on isolated T cells in this study, previous literature has reported that PVT1 is upregulated following T cell receptor activation (25) and participates in regulating T cell proliferation and Th1/Th17 differentiation (26). Therefore, the possibility that PVT1 acts within infiltrating T cells to enhance their effector functions cannot be ruled out. Future studies using T cell-specific PVT1 knockdown or overexpression, combined with single-cell RNA sequencing, should be conducted to identify the direct targets and signaling pathways of PVT1 within T cells.

CIRI typically triggers inflammatory cascades that disrupt blood–brain barrier integrity, enabling infiltration of peripheral immune cells (e.g., neutrophils, monocytes, macrophages and T cells), thereby exacerbating neuroinflammation (27). Although emerging evidence implicates the immune system in stroke pathogenesis, the mechanisms through which inflammatory cells and their molecular mediators drive CIRI-induced neuroinflammation remain poorly characterized. Accumulating evidence suggests that T cell subsets are pivotal in maintaining the homeostasis and functionality of the central nervous system (28). CD4 + T cells exhibit a significant increase within 24 h post-CIRI, peak between the third and fourth days, and can persist for extended periods. Research has demonstrated that CD4 + T cells stimulate multiple pro-inflammatory factors and participate in the pro-inflammatory cascade following stroke. The depletion of CD4 + T cells has been shown to markedly improve cognitive dysfunction in CIRI mice models (29), underscoring their role as key mediators of post-ischemic brain tissue damage. Furthermore, within the first month following CIRI, a substantial increase in CD4 + and CD8 + T cells has been observed in the peri-infarct region, indicating prolonged activation of these brain-invading T cells that contribute to neural repair (30). Previous studies have reported elevated PVT1 levels in peripheral blood samples from patients with IS, which are significantly correlated with neurological impairment (31). In the present study, we characterized the dynamics of PVT1 and T cells at different time points after MCAO within a transcriptomic context. Thus, our findings extend and complement previous research in this field.

Current anti-inflammatory treatments for CIRI generally lack cell specificity. PVT1 is markedly upregulated in the ischemic cerebral, and its knockdown attenuates T cell inflammatory infiltration, indicating that PVT1 could be a therapeutic target for CIRI. In our study, PVT1 expression was significantly positively correlated with pro-inflammatory cytokines such as IL-6 and IL-1β, suggesting that PVT1 levels may reflect the severity of T cell-related neuroinflammation. Clinically, PVT1 levels in peripheral blood mononuclear cells or extracellular vesicles from IS patients could be measured in the future to assess their association with post-stroke neurological deterioration, infection risk, and clinical outcomes.

However, this study has several limitations. First, deconvolution analysis of immune cell proportions may introduce systematic bias, which would require validation through higher-resolution approaches, such as single-cell RNA sequencing (32). Second, the absence of human CIRI brain transcriptome data prevents definitive conclusions regarding whether PVT1 shows conserved differential expression patterns and biological functions between rodent models and CIRI patients. Third, compared with mRNA studies, functional investigations of lncRNAs *in vivo* demands substantially higher knockdown efficiency due to their characteristically low abundance. Fourth, only male mice were used, which may not account for sex-dependent differences in post-stroke neuroinflammatory responses and thus may limit the generalizability of our findings. Our future studies will incorporate both sexes to better elucidate sex-specific mechanisms and to validate the generalizability of our findings. Emerging methodologies, including CRISPR-Cas9-mediated lncRNA knockout, may offer enhanced specificity and reduced off-target effects.

## 5 Conclusion

Through comprehensive bioinformatics analysis of whole-genome RNA-seq datasets, our study revealed that PVT1 was upregulated in CIRI and facilitated T cell activation in vivo. PVT1 knockdown demonstrates neuroprotective effects by mitigating brain ischemic injury induced neuroinflammation.

## Data Availability

The original contributions presented in the study are included in the article/Supplementary material, further inquiries can be directed to the corresponding authors.

## Footnotes

1
https://www.gencodegenes.org/

2
https://www.kegg.jp/kegg/kegg1.html

3
https://string-db.org

4
https://www.gsea-msigdb.org/

## Glossary

CIRI: Cerebral ischemia reperfusion injury
IS: Ischemic stroke
LncRNAs: Long non-coding RNAs
PVT1: Plasmacytoma variant translocation 1
MCAO: Middle Cerebral Artery Occlusion
GO: Gene Ontology
KEGG: Kyoto Encyclopedia of Genes and Genomes
GSEA: Gene Set Enrichment Analysis
BP: Biological process
CC: Cellular component
MF: Molecular function
qPCR: Quantitative polymerase chain reaction
ELISA: Enzyme-linked immunosorbent assay
TTC: 2,3,5-triphenyl tetrazolium chloride
BWC: Brain water content
HE: Hematoxylin–eosin
IF: Immunofluorescence
mNSS: Modified Neurological Severity Score
IL-6: Interleukin 6
IL-1β: Interleukin 1 beta
IL-4: Interleukin 4
IL-10: Interleukin 10
PCA: Principal component analysis
