# Supplementary material for: Identification and validation of neuroinflammation related lncRNA PVT1 with transcriptome-wide analysis in cerebral ischemia-reperfusion injury
Source: Front Neurol. 2026 Apr 29;17:1796559. doi: 10.3389/fneur.2026.1796559 (PMC13167490; doi:10.3389/fneur.2026.1796559)
Supplement: Supplementary file 1 [file Table_1.DOCX]

**Table S1** PCR primers sequences

| RNA | Forward sequence | Reverse sequence |
| --- | --- | --- |
| AI662270 | 5′-ACAACCACATCAAGAGGCAGAAC-3′ | 5′-TTCATGCAACTCACGGACCAAG-3′ |
| AU020206 | 5'-GGCACCAACGACTCCACCTG-3' | 5'-TGGCAGCGGATGAGGCATTG-3' |
| Gm20667 | 5’-AAACCATCTAAGAGACTGC-3’ | 5’-TTACCTTGACTGATCTTGTTTTGACA-3’ |
| PVT1 | 5'-CCTGGTGAAGCATCTGATGCACG-3' | 5'-GCCAGGCTTTGTGGCACACGC-3' |
| Mir142hg | 5'-GGAGCCTTGTCAGTATGGAAATC -3' | 5'-TGATGGCCCTATCCGATCCC-3' |
| GAPDH | 5'-AGCCACATCGCTCAGACAC-3' | 5'-GCCCAATACGACCAAATCC-3' |
